# Formulation for Effective Screening and Management of Nonalcoholic Steatohepatitis: Noninvasive NAFLD Management Strategy

**DOI:** 10.1155/2016/6343656

**Published:** 2016-06-13

**Authors:** Kanae Hirose, Tsutomu Kanefuji, Takeshi Suda, Souichi Sugitani, Keisuke Nagasaki, Tomoyuki Kubota, Masato Igarashi, Shuji Terai

**Affiliations:** ^1^Division of Gastroenterology and Hepatology, Graduate School of Medical and Dental Sciences, Niigata University, Niigata 951-8122, Japan; ^2^Department of Gastroenterology and Hepatology, Uonuma Institute of Community Medicine, Niigata University, Minami-Uonuma 949-7302, Japan; ^3^Division of Gastroenterology and Hepatology, Tachikawa Medical Center, Nagaoka 940-8621, Japan; ^4^Division of Homeostatic Regulation and Development, Graduate School of Medical and Dental Sciences, Niigata University, Niigata 951-8122, Japan; ^5^Division of Gastroenterology and Hepatology, Saiseikai Niigata Second Hospital, Niigata 950-1104, Japan

## Abstract

To establish a versatile means for screening and management of nonalcoholic steatohepatitis (NASH), shear wave velocity was measured in 20 normal controls and 138 consecutive nonalcoholic fatty liver disease (NAFLD) cases. Referencing biochemical properties in 679 healthy volunteers, a formula to distinguish NASH suspects was established and validated in another cohort of 138 histologically proven NAFLD cases. NASH and simple steatosis (SS) suspects were selected based on a plot of shear wave velocity against age. A formula consisting of five factors (*γ*-glutamyl transpeptidase, alkaline phosphatase, platelet counts, body mass index, and presence/absence of type 2 diabetes mellitus) distinguished NASH suspects from SS suspects with area under the receiver operating characteristic curve values of 86% and 84% in the development and validation cohorts. Among 25 NAFLD cases in which shear wave velocity was repeatedly measured, 8 and 9 cases revealed an increase or decrease, respectively, of shear wave velocity in the entire liver, and the corresponding change in shear wave velocity was primarily observed in the right lobe or the left lateral segment, respectively. These results suggest that the new formula and sequential shear wave velocity measurements at each segment enable high throughput screening of NASH suspects and noninvasive assessment of pathophysiological alleviation/aggravation in cases of NASH.

## 1. Introduction

Nonalcoholic fatty liver disease (NAFLD) is pandemic worldwide [1]. Within the broad spectrum of NAFLD, nonalcoholic steatohepatitis (NASH) is the most severe form because of its propensity to progress toward fibrosis of the liver, cirrhosis, and eventually hepatocellular carcinoma [2]. A histological evaluation in a biopsy specimen is currently the gold standard to confirm the presence of chronic necroinflammation and to evaluate the pathophysiological extent in NAFLD [3]. Unfortunately, however, it is impractical to conduct liver biopsies in a large population with NAFLD for the diagnosis of NASH and to repeat liver biopsies through the long clinical course of the disease. In the United States and other western countries, the prevalence of NAFLD is estimated to be as high as 30% [4]. Therefore, there is an urgent need to establish an efficient and practical method to identify NASH suspects in routine medical checkups and to encourage those patients to visit a hospital.

**Figure 1:**
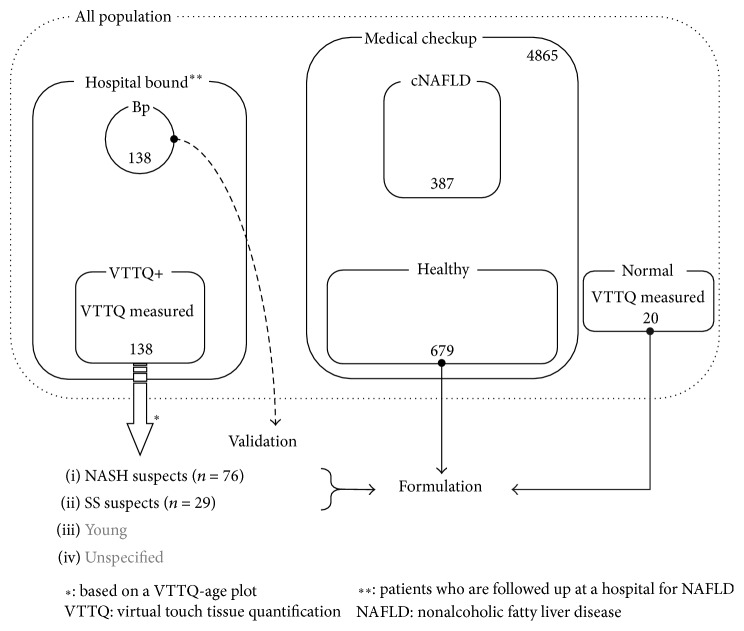
Study design.

Zhang et al. reported that, by detecting earlier stages of NAFLD and by implementing treatment according to current guidelines, surveillance demonstrates the potential to limit the transition of patients towards liver cirrhosis and end stage liver disease and its associated quality of life and economic costs [5]. In their surveillance strategy, transient elastography or virtual touch tissue quantification (VTTQ) was employed as an effective and interchangeable method for screening of the liver fibrosis. We also have reported the promising capability of VTTQ to quantify fiber accumulation and to infer the functional liver reserve in NASH [6]. It is not practical, however, to use a special modality or a nonroutine laboratory test to screen for NASH suspects in medical checkup fields. A scoring system with routine laboratory tests, such as BARD [7], is a promising alternative. However, the majority of such potential surrogate tests were developed to distinguish fibrous stages in NASH rather than distinguish NASH in NAFLD. The cohorts used in formulating those scores consist of cases that largely deviate from the population of routine medical checkup patients (Figure 1). For example, 58% of patients had a BMI between 30 and 39 in the cohort that was used to develop BARD score.

The aim of this study is to develop a formula that distinguishes NASH suspects in a broad range of population using routine laboratory tests. A cohort was grouped based on VTTQ values, which were measured in several cases again after a year or later. The efficacy of the formula was validated in another cohort including histologically proven NASH. The rationale of our formula and the significance of VTTQ measurement in NASH management are discussed.

## 2. Materials and Methods

### 2.1. Patients

The design of the present study is summarized in Figure 1. One hundred fifty-eight cases, consisting of 138 consecutive outpatients suffering from NAFLD (VTTQ+) and 20 normal controls (Normal), were subjected to VTTQ measurements. NAFLD was diagnosed based on the criteria proposed by the Asia-Pacific Working Party on NAFLD [8], and background characteristics are summarized in Table 1. In brief, fatty liver as observed by abdominal US was defined by the increased echogenicity of the liver along with the presence of any two out of the following three findings: liver-kidney contrast, vascular blurring, and deep attenuation of echo beam. Two expert pathologists independently evaluated liver biopsy specimens and judged fibrosis staging and inflammatory grading on the basis of Brunt criteria [9]. To evaluate the effects of aging on serum biochemical properties, 679 cases in the age range between 30 and 74 years were selected as healthy volunteers (Healthy) from 4865 medical checkup visitors who fulfilled all of the following requirements: negative reactions for anti-HCV antibody and HBsAg, less than 25 kg/m^2^ of BMI, normal ultrasonogram, less than 5.5% of HbA1c, no habitual alcohol intake, and less than 30 IU/L of alanine aminotransferase (ALT). Another 387 cases were selected as NAFLD in a cohort of medical checkup patients (cNAFLD), who were diagnosed with fatty liver by ultrasound and fulfilled all assumptions in “Healthy” except for normal ALT and HbA1c. An independent cohort consisting of 138 histologically proven NAFLD cases (Bp, 22 SS and 116 NASH cases) was used as the validation cohort. Informed consent was obtained from each patient from whom a VTTQ measurement was taken. The Niigata University Graduate School of Medical and Dental Sciences Human Research Committee, which did not require informed consent for a retrospective study using medical records or imaging examinations, approved the present study, which conformed to the ethical guidelines of the 2008 Declaration of Helsinki.

### 2.2. Biochemical and Immunohistochemical Analyses

Routine blood biochemical parameters were measured in the clinical laboratories of our hospitals. Type 2 diabetes mellitus (T2DM) was diagnosed based on the criteria of the Japan Diabetes Society [10], which defined T2DM as when hyperglycemia meets diabetic type criteria more than twice on separate days, or by a single plasma glucose test when one of the following three conditions exists: (1) the subject has typical symptoms of diabetes mellitus, (2) HbA1c is 6.1% or higher, and (3) there is unequivocal diabetic retinopathy. The criteria of diabetic type are as follows: fasting plasma glucose of 126 mg/dL or higher, plasma glucose 2 hours after 75 g glucose load of 200 mg/dL or higher, and/or casual plasma glucose higher than 200 mg/dL. Bone ALP was measured in the serum using a chemiluminescent enzyme immunoassay in *μ*g/L and was converted into U/L: U/L = (*μ*g/L + 2.265)/0.733, which was reported to show correlation coefficient of 0.962 [11].

### 2.3. Measurement of Shear Wave Velocity

VTTQ was measured using an ACUSON S2000 ultrasound system (Siemens Medical Solutions Inc., CA, USA). During a regular ultrasound observation, ROI is placed in each of the four segments: the lateral, medial, anterior, and posterior segments [12]. Based on our previous observations [6], VTTQ was measured three times in each segment and evaluated as a median of all four segments or in each segment. As for 25 NAFLD cases that consented to subsequent VTTQ measurements, the second VTTQ was measured 372 (interquartile range: 365–903) days after the first measurement.

### 2.4. Statistical Analyses

VTTQ was compared using a Kruskal-Wallis test among five histological fibrosis stages. A correlation between two metric variables was evaluated by calculating Spearman correlation coefficient. To estimate independent risk predictors for NASH, a binomial logistic regression analysis was used as a stepwise method with entry and removal limits of *p* < 0.05 and *p* > 0.10. The probability of NASH was calculated and coined as GAP-M by entering the coefficients that were calculated in the binomial logistic regression analysis as *β* of significant factors into the logistic regression equation. To judge the clinical usefulness, area under the receiver operator characteristic curve (AUROC) was calculated. Alteration of VTTQ values during sequential measurements was analyzed using Wilcoxon matched-pairs signed rank test. All statistical analyses were conducted with GraphPad Prism version 6.0 (GraphPad Software Inc., La Jolla, United States) or SPSS version 17.0 (SPSS Inc., Chicago, USA), and two-sided *p* values less than 0.05 were considered statistically significant.

## 3. Results

### 3.1. Shear Wave Velocity Increases with the Progression of Fibrosis Stage

**Figure 2:**
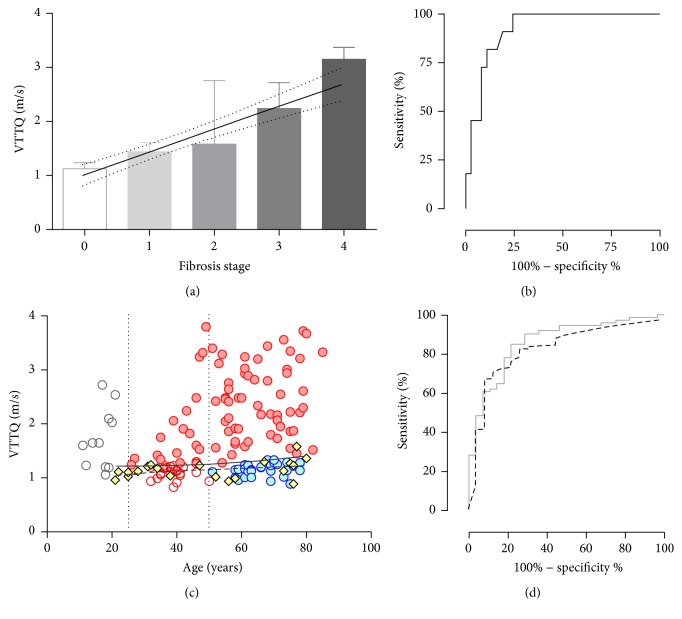
VTTQ and GAP-M discriminating NASH/SS suspects in NAFLD. (a) The median VTTQ and histological fibrosis stages (continuous line, best hit; dotted line, 95% confidence). (b) ROC of VTTQ distinguishing stages 0–2. (c) Four groups in VTTQ+ (see Figure 1, Normal (yellow)), Young (white), NASH suspects (red closed), SS suspects (blue), and Unspecified (red open) (continuous line, 99% confidence; dotted line, best hit). (d) AUROCs for GAP-M to distinguish NASH suspects from SS suspects (grey continuous line) or to distinguish histologically proven NASH from SS (black dotted line).

Fibrosis stages were histologically evaluated in 28 VTTQ+ cases, and VTTQ values for F0 were obtained from Normal, for which no histological evaluation was performed. The median VTTQ values in stages 0, 1, 2, 3, and 4 were 1.13 (1.02–1.24, *n* = 20), 1.44 (1.20–1.61, *n* = 6), 1.58 (1.06–2.76, *n* = 11), 2.24 (1.60–2.72, *n* = 7), and 3.15 (2.96–3.73, *n* = 4), respectively, and were significantly different as shown in Figure 2(a) (*p* < 0.0001). A post hoc test revealed significant differences between stages 0 and 3 (*p* < 0.01) and between stages 0 and 4 (*p* < 0.001). VTTQ value was significantly correlated with histological fibrosis stage (*p* < 0.0001, *r* = 0.72) and increased by 0.42 m/sec for one stage.

When the probability of discriminating between fibrosis stages 0–2 from other stages was evaluated using ROC analysis, VTTQ showed a sensitivity and specificity of 90.9% and 81.1%, respectively, with a cut-off value of the speed at 1.59 m/sec as shown in Figure 2(b). AUROC was 92.3% and was significant (*p* < 0.0001).

### 3.2. NAFLD Cases Can Be Divided into Subgroups Based on VTTQ Value and by Age

VTTQ was positively correlated with age in VTTQ+ cases in patients 11 to 85 years old and increased at a rate of 0.012 m/sec/year, as shown in Figure 2(c) (*p* = 0.0011, *r* = 0.27), but this correlation was not seen in Normal cases between the ages of 21 and 80 years (*p* = 0.088). Based on the plot, VTTQ+ was divided into four groups. Patients who were below 25 years old (Young, *n* = 11) showed an isolated distribution relative to other age groups. VTTQ values higher than the 99% confidence interval (CI) of the value in Normal cases were observed in 59.8% of the cases at 25 years of age or older, in which fiber accumulation was considered to have taken place in the liver (NASH suspects, *n* = 76). Although VTTQ is not likely to increase over the 99% CI with increasing age in the rest of the cases, one case that revealed a VTTQ of 1.05 m/sec at the age of 41, which was less than 99% CI, progressed to 2.48 m/sec after three years. In contrast, no case in patients over 50 years old surpassed the 99% CI during sequential measurements. Therefore, only elderly cases over the age of 50 with a VTTQ value less than the 99% CI were classified into suspects of simple steatosis (SS suspects, *n* = 29), and the others were categorized as Unspecified (*n* = 22).

VTTQ was significantly correlated with age in NASH suspects at a rate of 0.020 m/sec/year (*p* = 0.0013, *r* = 0.36), but not in SS suspects (*p* = 0.11). As shown in Table 2, univariate comparisons between SS and NASH suspects revealed significant differences in age, BMI, AST/ALT ratio, *γ*-glutamyl transpeptidase (*γ*-GTP), total bilirubin, Plt, and VTTQ value.

### 3.3. NASH Suspects Can Be Distinguished among NAFLD Patients at Medical Checkup Using a Scoring System

A total of 804 cases consisting of NASH and SS suspects and Normal and Healthy (Figure 1) cases were subjected to a binomial logistic regression analysis to distinguish NASH suspects from others. For this purpose, 13 variables were selected from the point of view of the availability in medical checkup fields and the general assumption of close association with metabolic syndrome and liver damage: age, gender, BMI, T2DM (yes/no), hypertension (yes/no), albumin, AST/ALT ratio, ALP, *γ*-GTP, total bilirubin, triacylglycerol, total cholesterol, and Plt. In the results, NASH suspects could be efficiently differentiated from others using a formula (GAP-M) consisting of five variables (Table 3):

(1) F=0.027×γ-GTP+0.01×ALP−0.251×Plt+0.496×BMI+2.043×T2DM yes,1;no,0−13.064GAP-M=exponentialF1+exponentialF.

**Figure 3:**
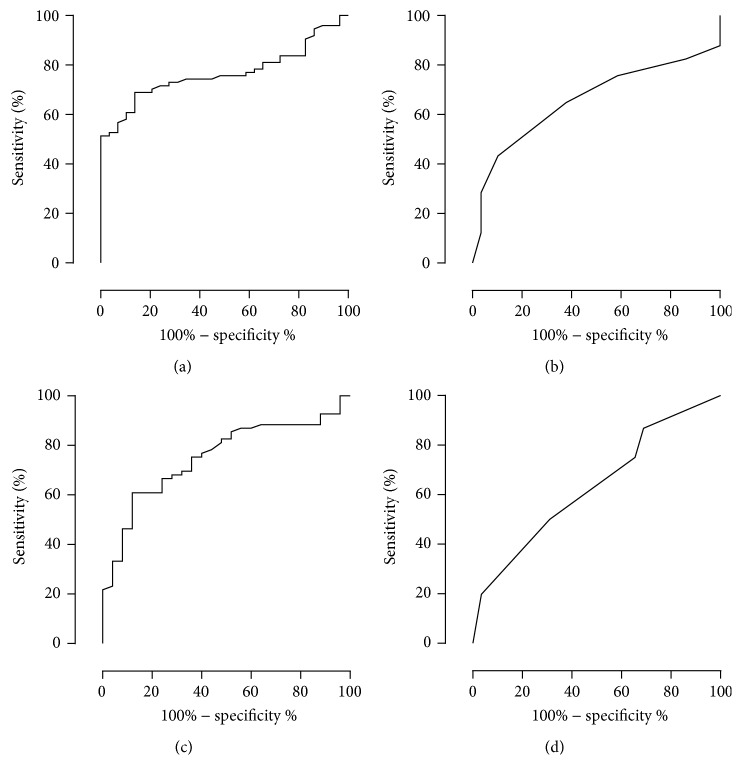
Efficacy of various scoring systems to differentiate NASH suspects in NAFLD. AUROCs distinguishing NASH suspects from SS suspects (see Figure 1) for Fib-4 index, AP index, NAFLD fibrosis score, and BARD score in the order of (a), (b), (c), and (d).

AUROC was calculated to assess the usefulness of GAP-M to distinguish NASH suspects from SS suspects, and the results were compared with those of other scoring systems for liver fibrosis. As shown in Figure 2(d), GAP-M revealed 86.4% of AUROC and distinguished NASH suspects from SS suspects with an accuracy of 79.4% (sensitivity: 90.5%; specificity: 71.4%) by adapting a cut-off value of 0.3. In contrast, the Fib-4 index, AP index, NAFLD fibrosis score, and BARD score showed AUROCs of 76.6%, 65.7%, 75.3%, and 63.3%, respectively, as shown in Figure 3. GAP-M judged 18.3% as NASH suspects in the cNAFLD group. In Bp, which consisted of 138 histologically proven cases with background characteristics shown in Table 1, GAP-M distinguished NASH from SS with an accuracy of 75.4% (sensitivity: 73.3%; specificity: 86.4%) with an AUROC of 84.1% (Figure 2(d)).

### 3.4. Longitudinal Measurements of VTTQ Reflect Pathophysiological Alterations in NASH

**Figure 4:**
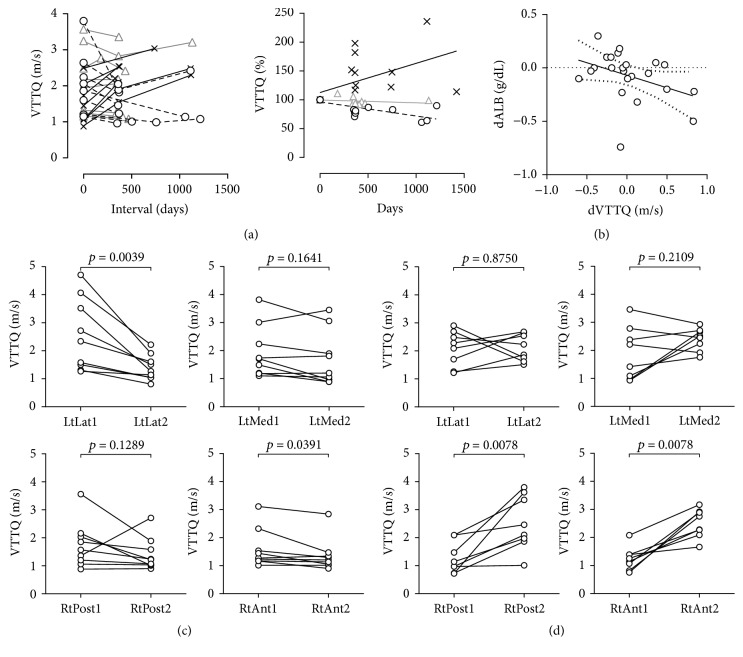
Characteristics of VTTQ in time and space during the course of NAFLD. (a) A plot of sequential measurements of VTTQ (median) in m/sec (left) or percentage to the first measurement (right). Cases with VTTQ getting smaller (circle), larger (cross), or stable (triangle). The lines of the right column reveal the best hit for each group (continuous for cross, dotted for circle, and grey for triangle). (b) The correlation between the alteration rates of VTTQ (dVTTQ) and serum albumin concentration (dALB) per year (continuous line, best hit; dotted line, 95% confidence). ((c) and (d)) VTTQ values of the first (1) and second (2) measurements at each segment: lateral (LtLat), medial (LtMed), posterior (RtPost), and anterior (RtAnt), in the cases with VTTQ of the entire liver getting smaller (c) or larger (d) in a follow-up period.

Next, the efficacy of VTTQ measurements over time was evaluated in 25 cases in which VTTQ and physiochemical markers were quantified. VTTQ increased by 0.23 m/sec/year in 8 aggravated cases, while VTTQ changed by −0.22 m/sec/year and −0.043 m/sec/year in 9 alleviated and 8 stable cases, respectively (Figure 4(a)). When VTTQ values were plotted as percent along the time course, the values were significantly correlated in each group (alleviated, *p* < 0.0001, *r* = −0.83; aggravated, *p* = 0.0002, *r* = 0.76; stable, *p* = 0.0055, *r* = 0.63). The alteration of VTTQ was significantly different among groups (*p* = 0.00018), and a post hoc test revealed that a significant difference existed between the aggravated and alleviated groups (*p* < 0.0001). Consistently, the alteration of albumin in a year showed a significant negative correlation with the VTTQ difference, as shown in Figure 4(b) (*p* = 0.047, *r* = −0.42). In addition, albumin increased by 0.1 (−0.075–0.3) g/dL in the alleviated group, while albumin decreased by −0.2 (−0.45–0.05) g/dL in the aggravated group. The difference between these two groups with regard to albumin approached statistical significance (*p* = 0.055).

### 3.5. VTTQ Is Unevenly Altered throughout the Liver during the Course of NASH

Next, VTTQ changes were evaluated in each segment. As shown in Figure 4(c), the 9 alleviated cases revealed a significant VTTQ reduction only in the lateral segment (*p* = 0.0039). The correlation of VTTQ and age was observed in the lateral segment (*p* = 0.042, *r* = −0.4834) but not in the other segments (*p* = 0.48, *p* = 0.38, and *p* = 0.43 for the posterior, anterior, and medial segments, resp.). In contrast, VTTQ was significantly increased in the right lobe involving both anterior and posterior segments in 8 aggravated cases (Figure 4(d), *p* = 0.0078 and *p* = 0.0078, resp.), but not in the left lobe of either the lateral or the medial segment (*p* = 0.88 and *p* = 0.21, resp.). VTTQ significantly differed and tended to be correlated with age in the anterior (*p* = 0.0005, *r* = 0.79) and posterior (*p* = 0.065, *r* = 0.47) segments, while there was no correlation in the left lobe (*p* = 0.22 and *p* = 0.78 in the medial and lateral segments).

## 4. Discussion

It is logical to assume that a scoring system that was formulated to distinguish advanced fibrosis in a NASH cohort may not be useful for screening for NASH in cases undergoing a medical checkup. AUROC values less than 80% indicated that Fib-4, AP index, NAFLD fibrosis score, and BARD are not suitable for the screening. In contrast, NAFIC [13] and HAIR [14] scores were developed in a manner to differentiate NASH in NAFLD; however, both scores require nonroutine laboratory tests such as C-peptide. Furthermore, the cohorts in whom the scores were formulated were noticeably different from the general population. The enrollment criteria for HAIR development required an adjustable gastric band and NAFIC score was formulated based on histologically proven cases. In contrast, NAFLD cases were subdivided into four groups in the present study based on a noninvasive examination of VTTQ measurement. Furthermore, normal control and healthy volunteers were involved to represent the general population. The significant correlations between age and NASH-related clinicopathological factors such as albumin were only observed in NASH suspects, not in SS suspects or Healthy cases (Supplementary Figure 1a, in Supplementary Material available online at http://dx.doi.org/10.1155/2016/6343656), which strongly suggest that NASH and SS suspects represent aggressive and stable cases in NAFLD. The differences between SS suspects and NASH suspects in univariate analyses further supported the pathophysiological progression in NASH but not in SS suspects. The GAP-M-developed cohort is considered to be less biased than those that have been used in the development of the score with respect to medical checkup visitors. The five constituents of GAP-M seem to be reasonable in estimating pathophysiological extent of metabolic syndrome (BMI and T2DM) and liver fibrosis (Plt, *γ*-GTP, and ALP), as *γ*-GTP was reported to be a surrogate marker of TNF-*α* expression in the liver [15] and ALP seems to be closely associated with osteopontin expression in the liver (Supplementary Figures 1b–1d). In the results, GAP-M leads to the accuracy of more than 75% in the validation and the NASH frequency of 18.3% in NAFLD, which is reported to be 10–20% in Japan [16].

There are serious concerns regarding the use of histological evaluation, such as sampling errors and diagnostic variability among observers [3]. The volume of tissue obtained by a needle biopsy is less than 1/50,000 of the entire liver, and biopsies are typically from a single site, leading to difficulty in discriminating adjacent fibrosis stages [17, 18]. Liver biopsy may not be able to be repeated over time. It has been suggested that many surrogate markers for liver fibrosis can distinguish the extended fibrosis stages and show similar AUROC values against histological diagnosis at a rate of approximately 85% [19]. As completely different types of markers possess similar sensitivity and specificity for the accumulation of fibrosis, it has been argued that the AUROC of surrogate markers may be restricted by the limitations inherent in histological evaluation itself [20]. By accounting for all of the abovementioned considerations, the significance of a new marker in NASH management should be evaluated not by referencing histology but by assessing clinical values for diagnosis, as a prognostic indicator, and in judgment of therapeutic efficacy. Only longitudinal evaluations using a noninvasive methodology are likely to draw an answer for these issues. For this purpose, TE has been widely applied in the clinic [21]; however, the target of TE is limited to a portion of the right lobe that is difficult to exactly locate on a corresponding area without real-time imaging in each study. On the other hand, VTTQ can precisely position a small ROI at a corresponding area over the liver in each measurement [6]. Because the pathophysiology progresses in a heterogeneous fashion through the liver [22, 23], it is crucial to sample SWV from a wide area of the liver and to precisely locate the target in sequential studies. Although a further study in a larger cohort is necessary to confirm the present findings, sequential VTTQ measurements have the potential to be effective in NASH management.

The concomitant analyses of fibrosis staging in histology and VTTQ measurements in NAFLD cases at various ages revealed that VTTQ increased by 0.42 m/sec as fibrosis was elevated by one stage and by 0.012 m/sec/year with increasing age, suggesting that it will take approximately 35 years to progress one fibrosis stage in our NAFLD cohort. The report of a similar observation suggested that NASH takes approximately 30 years to advance one fibrosis stage [24]. However, a NAFLD cohort consists of cases with a broad spectrum of diseases with differing rates of VTTQ increase among groups: 0.020 m/sec/year for NASH suspects and 0.22 m/sec/year for the aggravated cases along the sequential measurements. Therefore, the most aggressive cases may progress one fibrosis stage in a couple of years, and this observation is consistent with the report that approximately 30% of NASH will show fibrous progression in less than a half decade [25]. To efficiently find rapidly aggravating cases, our study suggested that VTTQ should be measured in the right anterior segment, in which VTTQ increased at a rate of 0.44 m/sec/year, which was almost double that in the entire liver. Reciprocally, the left lateral segment is suitable for detecting improvement, in which VTTQ decreased at a rate of 0.38 m/sec/year while the value decreased in the entire liver at a rate of 0.17 m/sec/year. The lobe selectivity on aggravation/alleviation may be due to different portal flow between the lobes. The blood from the superior mesenteric vein primarily flows into the right lobe, while the flow to the left lobe is largely dependent on perfusion from the spleen, according to the streamline theory [26]. The imbalance of the blood source in the portal vein could cause an uneven distribution of various chemicals, including nonesterified fatty acids, which are a potential culprit in NASH [27]. In terms of the left medial segment, the technical difficulty in visualizing the segment may explain the lack of VTTQ alteration. A single VTTQ measurement is useful in the diagnosis of advanced fibrosis stages, while serial measurements are likely to be sensitive enough to detect pathological alteration in a rapidly aggravated/alleviated NASH case. Physical studies targeting a specific section of the liver may miss the NASH pathological change in a short interval.

Limitations of our study include the relatively small number of cases and the selection bias. It has been reported that SWV is significantly slower in patients with SS compared with healthy volunteers [28]. Because no SS cases were involved in VTTQ measurements, the capability for NASH diagnosis in the early stages may be considerably lower than the actual potential of VTTQ measurements. Furthermore, the liver biopsies were not performed on the same day as VTTQ measurement, but after 16 (0–143) days. There is a possibility that the extent of steatosis and fibrosis had changed during that period. Our study design has the advantage that VTTQ was measured by one of 9 physicians chosen at random for each case. Thus, the data in this study include interobserver variation. As long as the physician has experience with standard B-mode ultrasound study, practically no special training appears to be required for VTTQ measurements.

## 5. Conclusions

A balanced diet and regular physical exercise have a powerful potential to improve NASH [29], and the digital presentation of improvement or deterioration in NASH is a strong incentive and motive for patients to follow an energy-balanced lifestyle. VTTQ would realize a four-dimensional (three-dimensional space plus time) evaluation of liver pathophysiology, leading to a better understanding of the pathogenesis and effective treatment by educating and encouraging NASH patients. GAP-M screening followed by sequential VTTQ measurements in liver segments is a promising means for a systematic approach in NAFLD management.

## Supplementary Material

Supplementary Figure 1a; alteration of serum albumin concentration with age.Supplementary Figure 1b-1d; association of ALP with OPN, which is suggested to play a key role in functional and histological progression of NASH.

## Figures and Tables

**Table 1:** Background characteristics.

	VTTQ+		Bp	
	Median	IQR	Median	IQR
Age (years)	58	40–69	60	41–69
Gender (male : female)	67 : 71		59 : 79	
Body mass index (kg/m^2^)	26.0	23.4–29.0	26.6	23.2–29.6
T2DM (yes : no)	50 : 88		40 : 98	
Hypertension (yes : no)	55 : 83			
Albumin (g/dL)	4.4	4.0–4.6		
ALT (IU/L)	43	28–81		
ALP (IU/L)	261	196–332	267	212–356
*γ*-GTP (IU/L)	60	35–115	61	44–110
Platelet count (×10^9^/L)	186	140–236	200	166–258
VTTQ (m/sec)	1.56	1.19–2.41		

IQR: interquartile range; T2DM: type 2 diabetes mellitus; ALT: alanine aminotransferase; ALP: alkaline phosphatase; GTP: glutamyl transpeptidase; VTTQ: virtual touch tissue quantification; VTTQ+: hospital bound cases which were suffering from nonalcoholic fatty liver diseases and subjected to VTTQ measurement; Bp: hospital bound cases which were suffering from nonalcoholic fatty liver diseases and subjected to liver biopsy.

**Table 2:** Univariate comparison between SS and NASH suspects.

	SS suspects		NASH suspects		Probability
	Median	IQR	Median	IQR	
Age (years)	66	61–72	59	50–72	0.014
Gender (male : female)	12 : 17		28 : 48		0.67
Body mass index (kg/m^2^)	24.4	22.9–26.3	26.8	24.4–30.5	0.0016
T2DM (yes : no)	10 : 19		36 : 40		0.23
Hypertension (yes : no)	14 : 15		36 : 40		0.93
Albumin (g/dL)	4.4	4.2–4.6	4.3	3.9–4.5	0.13
ALT (IU/L)	37	21–55	43	27–82	0.19
AST/ALT	0.80	0.60–1.15	1.17	0.74–1.54	0.011
ALP (IU/L)	252	202–290	292	202–365	0.14
*γ*-GTP (IU/L)	46	24–104	60	40–126	0.014
Total bilirubin (mmol/L)	12.9	10.3–18.8	15.4	13.7–20.5	0.032
Triacylglycerol (mmol/L)	1.43	0.84–1.80	1.31	0.98–1.82	0.76
Total cholesterol (mmol/L)	5.14	4.72–5.59	4.73	4.16–5.40	0.085
Platelet count (×10^9^/L)	207	191–264	159	115–194	<0.0001
VTTQ (m/sec)	1.15	1.07–1.24	2.20	1.67–2.92	<0.0001

SS: simple steatosis; NASH: nonalcoholic steatohepatitis; IQR: interquartile range; T2DM: type 2 diabetes mellitus; AST: aspartate aminotransferase; ALT: alanine aminotransferase; ALP: alkaline phosphatase; GTP: glutamyl transpeptidase; VTTQ: virtual touch tissue quantification.

**Table 3:** Multivariate analysis.

In the equation			Not in the equation	
	*β*	Significance		Significance
*γ*-GTP	0.027	0.000	Age	0.081
ALP	0.010	0.001	Gender	0.375
Platelet	−0.251	0.000	Hypertension	0.464
BMI	0.496	0.000	Albumin	0.611
T2DM	2.043	0.001	Triacylglycerol	0.097
Constant	−13.064	0.000	Total cholesterol	0.054
			AST/ALT	0.704

AST: aspartate aminotransferase; ALT: alanine aminotransferase; GTP: glutamyl transpeptidase; BMI: body mass index; T2DM: type 2 diabetes mellitus; ALP: alkaline phosphatase.
