# Supplementary material for: Formulation for Effective Screening and Management of Nonalcoholic Steatohepatitis: Noninvasive NAFLD Management Strategy
Source: Gastroenterol Res Pract. 2016 Jun 13;2016:6343656. doi: 10.1155/2016/6343656 (PMC4921726; doi:10.1155/2016/6343656)
Supplement: Supplementary file 1 — Supplementary Figure 1a; alteration of serum albumin concentration with age.Supplementary Figure 1b-1d; association of ALP with OPN, which is suggested to play a key role in functional and histological progression of NASH. [file 6343656.f1.pdf]

Supplementary Figure 1

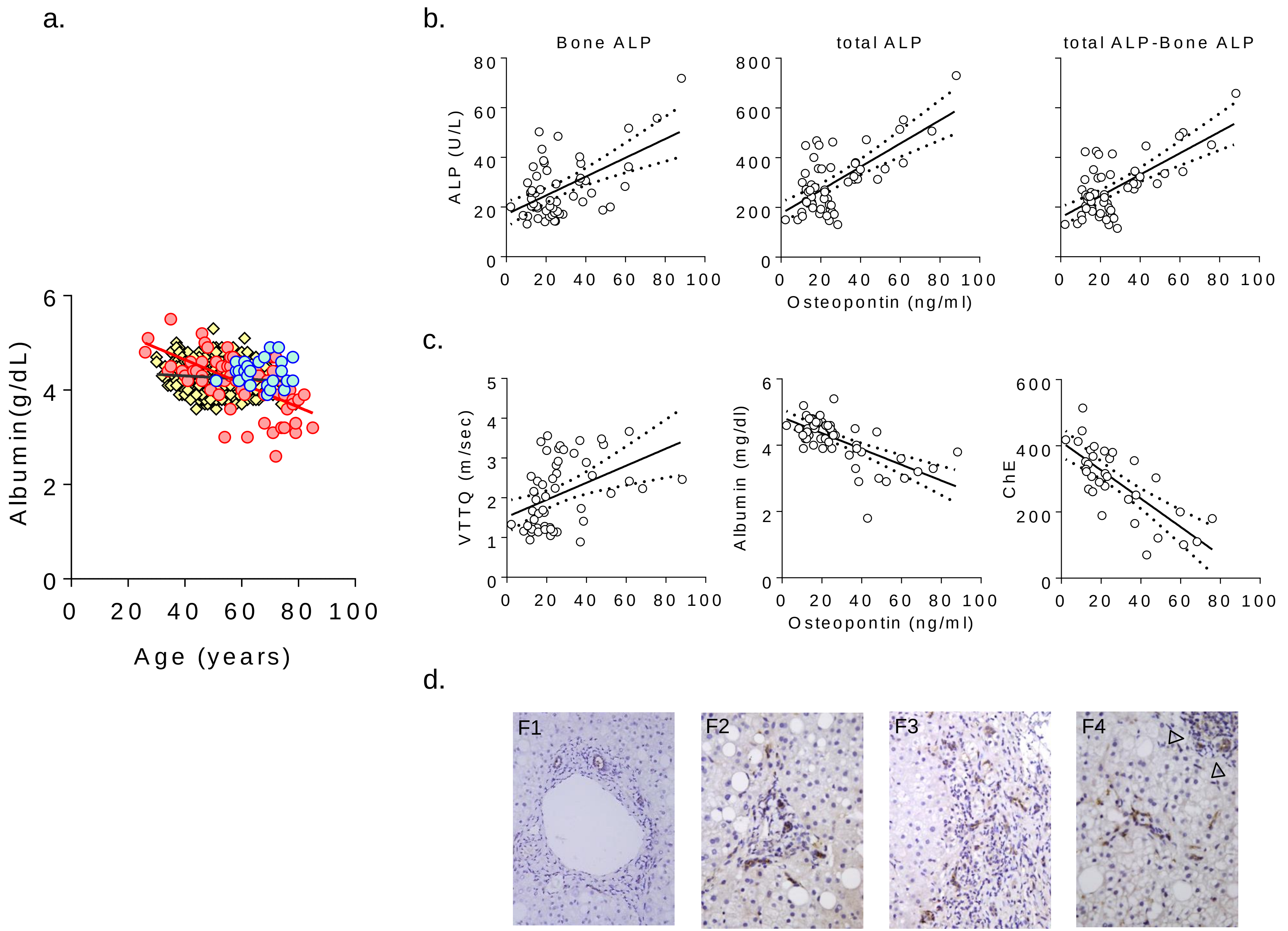

Supplementary data

Figure 1

a. To deduce if liver pathophysiology progressed with age specifically in NASH suspects, the alteration of albumin with age was compared between NASH suspects and Healthy or SS suspects. The serum albumin concentration showed a significant negative correlation with age in NASH suspects (red, p<0.0001, r=-0.62), but not in Healthy (yellow, p=0.15) or SS suspects (blue, p=0.87). The average reduction rate of albumin in NASH suspects was 0.025 mg/dL/year. The black and red lines are the best hit lines for Healthy and NASH suspects. **b and c.** To explore the relationship between ALP and fibrosis progression in NASH cases, a correlation between osteopontin (OPN) and ALP was investigated. The serum OPN concentration was quantified using an osteopontin human ELISA kit (Abcam 100618, Tokyo, Japan). OPN was significantly correlated with not only bone ALP (p=0.047, r=0.27) but also total ALP (p=0.0006, r=0.45). Furthermore, the Spearman correlation coefficient was substantially higher in the correlation with total ALP after subtraction of bone ALP (p=0.0005, r=0.46). Furthermore, serum OPN level was positively correlated with VTTQ (p<0.0001, r=0.59) and inversely correlated with functional liver reserve such as albumin (p<0.0001, r=-0.60) and cholinesterase (p<0.0001, r=-0.739). **d.** OPN expression in the liver was evaluated by

immunohistochemistry using anti-OPN antibody AF1433 (R&D Systems, Inc., Minneapolis, USA). The cells expressing OPN were scarcely detected in the liver at F1 stage and then increased in frequency at ductular reaction as fibrosis progressed from F2 to F3. In the F4 stage, the positive cells migrated into the hepatic lobule apart from the fibrous septa of ductular reaction (arrowheads).
